# Detection of virulence genes among *Staphylococcus saprophyticus* isolated from women with urinary tract infections: first report from Iran

**DOI:** 10.1186/s13104-023-06481-1

**Published:** 2023-09-11

**Authors:** Maryam Rafiee, Ezzat Allah Ghaemi

**Affiliations:** Laboratory Sciences Research Center, Golestan University of Medical Sciences, Gorgan, Iran

**Keywords:** *Staphylococcus saprophyticus*, Urinary tract infections, Biofilm, Virulence, Polymerase chain reaction

## Abstract

**Objective:**

The purpose of the present study was to investigate the biofilm production, and the presence of virulence genes and biochemical characteristics among the *S. saprophyticus* clinical isolates. A total of 35 clinical isolates of *S. saprophyticus* were collected from patients referred to several hospitals. By the crystal violet staining method, the capability of biofilm formation was performed. The genes associated with surface of *S. saprophyticus* were investigated by the PCR-sequencing techniques. Hemagglutination and lipase activity assays were also performed.

**Results:**

The results of crystal violet staining assay showed that 32 isolates (91%) form biofilm. Moreover, seven (20%), 13 (37%), and 12(34%) isolates were categorized as weak, moderate, and strong biofilm producers, respectively. virulence genes including UafA, Aas and Ssp had an overall prevalence of 88%, 91% and 80%, respectively. None of the isolates exhibited lipolytic activities. Regarding hemagglutination properties, only 11 (31%) isolates demonstrated hemagglutination of sheep erythrocytes. The results of this study indicate a high prevalence of UafA and Aas genes that can enhance the pathogenicity of *S. saprophyticus*, and Identification and better understanding of the functions of these genes can be used for therapeutic purposes. Maybe in the future we will be switch to anti-adhesion therapy because of drug resistance.

## Introduction

Urinary tract infections (UTI) are one of the most common bacterial infections in young and sexually active women [1]. An estimated 40–50% of women experience at least one UTI in their lifetime [2]. The costs associated with healthcare for UTIs annually exceed $ 1 billion in the USA [3].

*Staphylococcus saprophyticus* is a gram-positive, novobiocin-resistant, and coagulase-negative coccus that is the second most common etiological agent of UTI after *Escherichia coli* infection. *S. saprophyticus* is responsible for 5–20% of community-acquired UTIs in young women. Among *staphylococcal* species, *S. saprophyticus* is typically uropathogenic and can adhere to uroepithelial cells [2].

The distribution of virulence factors among isolates of *staphylococcal* species such as *S. aureus* and *S. epidermidis* has been extensively studied. However, few studies have investigated the presence of virulence genes in *S. saprophyticus*. In this study, we evaluated the known virulence factors among urinary tract isolates of *S. saprophytius*, including four surface protein genes and biofilm, lipase, and hemagglutinin production [2].

To date, six surface proteins have been characterized and described in *S. saprophyticus* colonizing the urinary tract. Among them, four cell wall-anchored proteins, featuring a conserved characteristic C-terminal LPXTG motif, have been identified in *S. saprophyticus*: The uro-adherence factor A (*UafA*) is a hemagglutinin which mediate adherence to the human bladder epithelial cells found in *S. saprophyticus* strains. *UafA* was the first protein to be classified as MSCRAMM in *S. saprophyticus*, suggesting that it plays an important role in adherence to the urinary tract, which is a challenging environment for bacterial colonization owing to urine flow. *S. saprophyticus* has another uro-adherence factor, plasmid-encoded *UafB*, found in ~ 5% of strains examined, which binds fibrinogen and fibronectin, which attach them to human bladder epithelial cells. The surface protein F of *S. saprophyticus* (*SssF*) is highly conserved among *S. saprophyticus* strains, and plays a role in resistance to linoleic acid present in the perineum section of the human gastrointestinal tract, but is not involved in uropathogenesis. *SdrI* is a cell wall surface protein found in a minority of isolates that can bind to collagen, and is the second surface protein carrying the LPXTG motif in *S. saprophyticus*. Protein surface-associated lipase (*Ssp*) is found in > 90% of isolates and is very important for infection in a murine model. However, the mechanism of action of *Ssp* in vivo remains unclear. *Aas*, which is conserved in nearly all *S. saprophyticus* isolates, is a multifunctional protein that has adhesive and autolytic properties, in addition to binding to fibronectin [2, 4].

In addition to cell wall-associated proteins, two cytoplasmic enzymes, urease and D-serine deaminase (DsdA), are crucial for *S. saprophyticus* survival in the bladder environment. D-serine present in urine is toxic to most urinary bacteria; however, some bacteria, such as *S. saprophyticus*, can grow in the presence of high concentrations of D-serine because of the DsdA enzyme. Urease production by *S. saprophyticus* is essential for efficient colonization of the bladder and kidneys, and is also associated with the formation of urinary infectious stones [5, 6].

It is estimated that up to 65% of bacterial infections are caused by biofilms. Therefore, it plays a significant role in infections, especially medical device-associated urinary tract infections (UTIs). Biofilm formation is now recognized as an essential virulence factor in several *Staphylococcus* species such as *S. saorophyticus* [7].

To the best of our knowledge, this is the first study to investigate the distribution of putative virulence factors of *S. saprophyticus* in Iran. This study aimed to evaluate the presence of these genes, their surface properties, and biofilm formation in *S. saprophyticus* strains that cause UTIs in women in Gorgan, Iran.

## Materials and methods

### Bacterial isolation and identification

This descriptive study was performed on thirty-five confirmed *S. saprophyticus* isolates collected from clinical specimens of patients with UTI in hospitals and laboratories in Gorgan, Iran, between May 2018 and September 2020. The isolates were confirmed by biochemical and PCR amplification for the *16SrRNA* assay, and antibiotic susceptibility was determined using the Kirby-Bauer method [8]. Biochemical features, such as lipase production, biofilm formation, hemagglutinin presentation, and the presence of four adherence factor genes in all *S. saprophyticus* isolates were evaluated in this study.

### Lipase activity assay

The lipolytic activity of *S. saprophyticus* isolates was assayed using a Baird-Parker agar plate (Merck Co., Germany). The isolates were streaked and a clear halo around the growth after incubation at 37 °C for 48 h was considered lipolytic activity [9].

### Hemagglutination

Hemagglutination was assessed, as previously described. Briefly, bacteria were grown for 16 h at 37 °C in Muller-Hinton broth. The cells were then washed twice with NaCl. The bacterial suspensions were adjusted to an optical density of 1.0 at OD600 nm. An erythrocyte suspension (2% in PBS) was added to an equal volume of the washed cells in a 96-well microtiter plate. The results were read after 2 h of incubation at room temperature. Positive hemagglutination appeared as homogeneous turbidity and a uniform thin film of erythrocytes coated the well, whereas negative hemagglutination appeared as a sink at the bottom of the well [10].

### Biofilm formation assay

Biofilm formation by the isolates was assessed using the microtiter plate method. An overnight culture of bacterial isolates was adjusted to 0.5 McFarland, diluted in TSB-glucose(1:100), and 200 µl aliquots were added to the wells of a 96-well plate and incubated for 24 h at 370 C. TSB-glucose was used as the negative control in the biofilm formation assay. After incubation, the plates were washed three times with 200 µL of sterile phosphate-buffered saline (PBS) to remove unattached bacteria. The adherent cells in each well were fixed with 99% methanol for 10 min and the plates were allowed to dry. The wells were stained with 200 µL 0.1% crystal violet (CV) for 5 min at room temperature. Stains were rinsed with water and the plates were allowed to dry. The stain was dissolved in 200 µL of 95% ethanol, placed in a shaker for 30 min, and the optical density was measured at an OD of 595 nm. The isolates were classified into four groups: (OD < ODc), no biofilm producer; (ODc < OD < 2 × ODc), weak biofilm producer; (2 × ODc < OD < 4 × ODc), moderate biofilm producer; and (4 × ODc < OD), strong biofilm producer. All experiments were performed in triplicate [11].

### PCR assay

The presence of virulence factor genes *Aas*, *Ssp*, *sdrI*, and *UafA* in *S. saprophyticus* isolates was investigated by PCR using specific primers. The primers described by Paiva-Santos et al. were used to amplify the *UafA* and *SdrI* genes [12]. The other primers for the amplification of ssp. and *Aas* were designed using free Primer3 software (Table 1). PCR mixture was performed in a final volume of 25 µl and contained 12.5 µl of 2× master mix (Ampliqon A/S, Odense, Denmark), including 1× PCR buffer, 0.4 mmol/L dNTPs, 3 mmol/L MgCl2, and 0.08 IU Taq polymerase; 1 µl of 10 pmol of each primer; and 9.5 µl sterile distilled water. A small amount of a colony was picked directly from the plate using a sterile tip and placed in a PCR tube [13]. All four genes were amplified under the following thermal conditions: initial denaturation at 95 °C for 5 min, followed by 30 cycles of denaturation at 95 °C for 45 s, annealing temperature at 51–60 ºC depending on the relevant genes for 30 s, extension at 72 °C for 30 s, and a final extension at 72 °C for 5 min. The PCR products were electrophoresed on 2% agarose gel and visualized using DNA Safe dye (Thermo Fisher). They were then photographed and analyzed under UV light after running at 100 V for 1 h. The purified PCR product from each positive sample was sequenced by Sanger sequencing (Macrogen Co., South Korea). Nucleotide sequences were analyzed using Chromas version 1.45. In addition, sequence comparisons were performed using the Nucleotide BLAST program and deposited in GenBank (https://www.ncbi.nlm.nih.gov/genbank/).

**Table 1:** The specific primers used for the detection of virulence genes among *S. saprophyticus* strains

Gene	Primer (5’ → 3’)	Amplicon size(bp)	T_m_ (°C)	reference
*UafA*	F-GTAGATGACTCCGTGGTTGAAG R-AGCGATTGTTCTCCCATTAGC	125	57	(12)
*SdrI*	F-CAACGTGCAACAACAGATGAC R-TATTTGATGGCGACGGAGTG	111	60	(12)
*Ssp*	F-ATTTATCATACCTTTCACGAGC R-GTTACTTGCCAGACACCTT	358	51	This study
*Aas*	F-GGCACTTTAATTGGTTGGGTA R-CTTGGCGTCGTAGATGGT	287	56	This study

### Statistical analysis

All data were analyzed using the GraphPad prism 8.3.1 software. A chi-square test was performed to determine the association between the biofilm formation phenotype and virulence genes. Statistical significance was set at p < 0.05.

## Results

### Biofilm formation

In this study, biofilm formation by 35 *S. saprophyticus* isolates was assessed using the microtiter plate method. The results demonstrated that 32(91%) of the isolates were biofilm producers; among them, 12 (34%) isolates showed strong biofilm formation, 13 isolates (37%) produced moderate biofilm, and seven isolates (20%) produced weak biofilms, whereas three isolates (8%) could not form a biofilm.

### Lipolytic activity

**Fig. 1:**
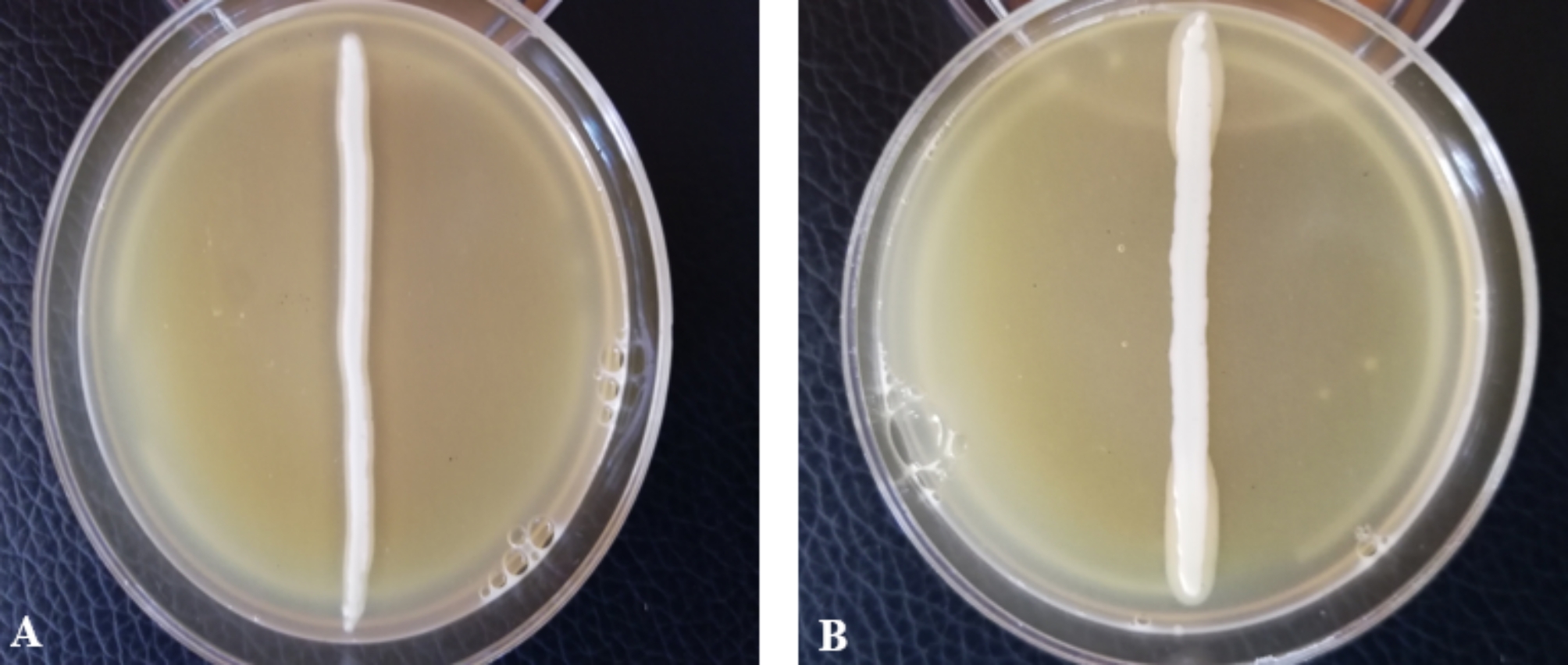
Lipase test result interpretation. **(A)**. Positive Lipase test: clear zone around the *S.aureus* ATCC 25,923 growth. **(B)**. The absence of a clear zone around the *S. saprophyticus* growth

Phenotypic detection of lipolytic activity in *S. saprophyticus* isolates was investigated (Fig. 1). None of the clinical isolates showed lipolytic activity on Baird-Parker agar plates, and no clear halos were observed after incubation at 37 °C for 48 h.

### Hemagglutination

Hemagglutinating properties were studied as previously described. Hemagglutination was observed in a minority of the *S. saprophyticus* strains. Of the 35 isolates, 11 (31%) demonstrated hemagglutination of sheep erythrocytes.

### Molecular detection of virulence factors

PCR amplification of virulence genes (*Aas*, *Ssp*, *UafA*, and *SdrI*) was performed on 35 clinical isolates. The results of the PCR assay showed that the most frequent gene was *Aas* (91%), followed by *UafA* (88%), and *Ssp* (80%). Another surface protein, *SdrI*, was not detected in any of the isolates. To confirm the amplified adhesin genes, PCR products of some isolates were sequenced and read using the Chromas software. The strain sequences were deposited in GenBank under accession numbers OP696966, OP696967, and OP973206 (https://www.ncbi.nlm.nih.gov/genbank/). Most isolates harbored adhesin genes, either singly or in combination. As shown in Table 2, five virulence gene profiles (A-E) were found, which profile “D” (*Aas*+, *Ssp*+, *UafA*+, *Sdrl*^−^) was the most frequent pattern, accounting for 71.4% (n = 25) of all isolates. In contrast, group “E” accounted for 2.8% (n = 1) of isolates with no virulence genes.

**Table 2:** Distribution of the virulence genes among *S. saprophyticus* isolates

Group	Virulence genes expression profile	No. (%) of isolates
A	*Aas+, Ssp*^*+*^_,_*UafA*^*-*^, *Sdrl*^*-*^	3 (8.5%)
B	*Aas+, Ssp*^*-*^_,_*UafA*^*+*^, *Sdrl*^*-*^	4 (11.4%)
C	*Aas*^*-*^_,_*Ssp*^*-*^_,_*UafA*^*+*^, *Sdrl*^*-*^	2 (5.7%)
D	*Aas*^*+*^_,_*Ssp*^*+*^_,_*UafA*^*+*^, *Sdrl*^*-*^	25 (71.4%)
E	*Aas*^*-*^_,_*Ssp*^*-*^_,_*UafA*^*-*^, *Sdrl*^*-*^	1 (2.8%)

### Correlation of phenotypic and genotypic virulence factors

**Fig. 2:**
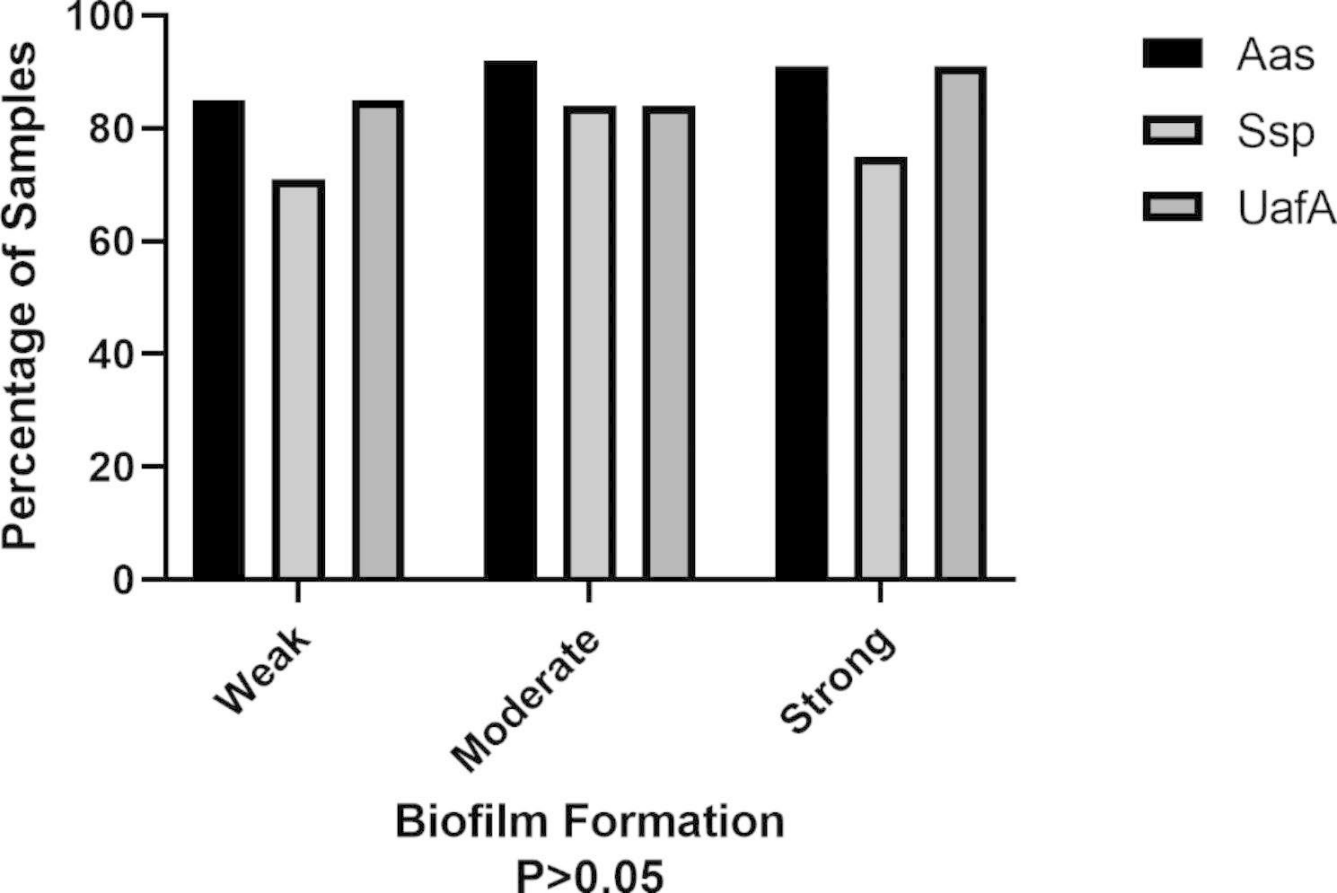
Comparison between biofilm formation and frequency of virulence genes in *S. saprophyticus* isolates

Overall, 32(91%) of the *S. saprophyticus* isolates formed biofilms. Likewise, there was no significant relationship between biofilm formation intensity (strong, moderate, and weak) and frequency of the virulence genes studied in *S. saprophyticus* isolates (p > 0.05) (Fig. 2).

## Discussion

UTIs account for more than 30% of hospital-acquired infections [14]. Most UTIs are biofilm-associated infections in which pathogenic bacteria colonize the urinary tract and medical devices such as indwelling catheters [7]. *S. saprophyticus* causes uncomplicated urinary tract infections, but there are few studies on its ability to produce biofilm [15]. According to the results of this study, a significant number of *S. saprophyticus* isolates (91%) could form biofilms. Biofilm production by *S. saprophyticus* isolates varies in different studies. According to Lawal et al., approximately 91% of the isolates were biofilm producers, which is similar to our findings [16]. In contrast, Martin et al. and Hashemzade et al. reported that 70% and 63% of these strains, respectively, were biofilm producers [15, 17]. These variations in the rates of biofilm formation may be due to the diversity of geographic areas or variations in antibiotic resistance.

Uropathogenic bacteria have a wide range of virulence factors attached to their cell surface. These adhesive proteins mediate binding to uroepithelial cells and tissue receptors, suggesting that UTI treatment should focus more on bacterial adhesion, which is essential for the initial attachment [2, 4]. At least six adhesins have been associated with urinary tract colonization by *S. saprophyticus*, and the presence of four surface protein genes, *Aas*, *UafA*, *Sdrl*, and *SsP*, in *S. saprophyticus* was assessed in this study.

Aas has hemagglutinin properties, binds to human ureters, and plays a role in colonization of rat kidneys in vivo [18]. This study showed that (91%) of the isolates possessed the *Aas*. This result is similar to the findings of previous studies conducted by Paiva-Santos et al., Al-Waeely et al., and Kleine et al., who found the Aas gene in all *S. saprophyticus* isolates [2, 12, 19]. In contrast, Alo et al. found the *Aas* gene in only 30% of *S. saprophyticus* isolates [20].

Uro-adherence factor A (*UafA*) plays an important role in the hemagglutination activity of *S. saprophyticus* and is associated with adherence to bladder epithelial cells [21]. In this study, the frequency of *UafA* was (88%). Paiva-Santos et al., Al-Waeely et al., and Kleine et al. reported the presence of this gene in all the isolates [2, 12, 19]. In contrast, Alo et al. showed that *UafA* was not detected in any of these isolates [20].

Hemagglutination ability in relation to adherence properties. The hemagglutination ability of *S. saprophyticus* is strongly related to *UafA* and *Aas* genes [10, 22]. Although most *S. saprophyticus* isolates in this study possessed these genes, only a few showed hemagglutination, implying that hemagglutination of this bacterium requires other factors.

The surface-associated protein of *S. saprophyticus* (*Ssp*) was identified as a surface-associated lipase that forms a fuzzy surface layer on bacteria [23]. Approximately 80% of the *S. saprohyticus* isolates in this study harbored the *Ssp* gene, which is similar to the findings of Kleine et al. [2]. (86%). Paiva-Santos et al. and Al-Waeely et al. shows that all isolates carried the *Ssp* gene [12, 19]. In contrast, Alo et al. detected *Ssp* in only eight (9%) of the isolates [20]. Although *Ssp* plays a role in the lipolytic activity of *S. saprophyticus*, none of the clinical isolates examined in this study exhibited lipolytic activity. The lipolytic activity of *S. saprophyticus* was studied by Kleine et al., who reported lipolytic activity in 66% of isolates [2]. However, the reason for this difference remains unclear. The ability of *Ssp* to express or translate its enzymatic activity correctly remains unclear and requires further investigation.

*SdrI* is another cell wall-associated protein belonging to the serine aspartate repeat protein family, which binds to collagen [24]. Previous studies have confirmed that this protein is not required for the initial colonization of *S. saprophyticus* in the urinary tract but is essential for persistence in the bladder and kidney [25]. In our study, *SdrI* was not detected in any isolate. This result is in agreement with the results of Alao et al. and Pavia et al. [12, 20]; however, in the studies by Kleine et al. and Alweely et al., the *SdrI* protein was found only in a minority of *S. saprophyticus* strains [2, 19].

## Conclusion

Among adherence-related genes, *UafA* and *Aas* were found in most urinary tract isolates of *S. saprophyticus*. These genes are related to the hemagglutination phenotype, but only a few isolates showed hemagglutination in vitro, suggesting that *S. saprophyticus* uses other gene products for hemagglutination, which requires further study.

### Limitations

A limitation of this study may be the lack of evaluation of expression levels of virulence-associated genes by quantitative real-time PCR, an approach that may help assess the role of each corresponding gene in UTIs infections. In addition, the lack of samples from various geographical locations in Iran is also a limitation of this study.

## Data Availability

The datasets generated during and or analysed during the current study are available from the Maryam rafiee ( sefidrooze@yahoo.com) on reasonable request.
